# High-frequency Ultrasound Reveals Epidermal-dermal Structural Alterations in an Experimental Model of Epidermolysis Bullosa

**DOI:** 10.2340/actadv.v106.44438

**Published:** 2026-02-17

**Authors:** Karl WALLBLOM, Artur SCHMIDTCHEN, Manoj PUTHIA

**Affiliations:** 1 Division of Dermatology and Venereology, Department of Clinical Sciences Lund, Lund University, Lund; 2 Department of Dermatology, Skåne University Hospital, Lund, Sweden

Epidermolysis bullosa (EB) comprises a group of rare genetic disorders characterized by painful blistering triggered by minimal mechanical trauma (1). Current diagnostic methods have limitations; histopathology is invasive and unsuitable for frequent monitoring, whereas clinical assessments may lack objectivity and sensitivity to detect subtle structural changes in the skin.

High-frequency ultrasound (HFUS) uses probes with frequencies above 15 MHz to visualize and quantify skin structures noninvasively. HFUS has demonstrated utility in monitoring various dermatological conditions, including inflammatory disorders, tumours, and scars (2). Notably, HFUS can visualize and quantify the subepidermal low-echogenic band (SLEB), with increased thickness correlating with heightened inflammatory activity in the skin (2–6).

To address the need for objective and noninvasive monitoring tools for EB, this study aimed to establish and validate an HFUS protocol to longitudinally monitor spontaneous, disease-driven EB-related skin changes in a junctional epidermolysis bullosa (JEB) murine model (*LAMC2^jeb^*) (7).

## MATERIALS, METHODS and RESULTS

Animal experiments were performed in accordance with the Swedish Animal Welfare Act SFS 1988:534 and approved by the Animal Ethics Committee of Malmö/Lund, Sweden. The B6.129X1-*LAMC2^jeb^*/DcrJ mouse strain (Stock #025467, Jackson Laboratory), which models non-Herlitz JEB, was used (7). Homozygous mice develop progressive cutaneous fragility with spontaneous blistering and ulceration visible on the tail, ears, and paws from 13–14 weeks of age (7). For HFUS assessment, we focused on the tail, as ears and paws were unsuitable due to their very small and irregular shape, thin skin, and curvature.

**Fig. 1:**
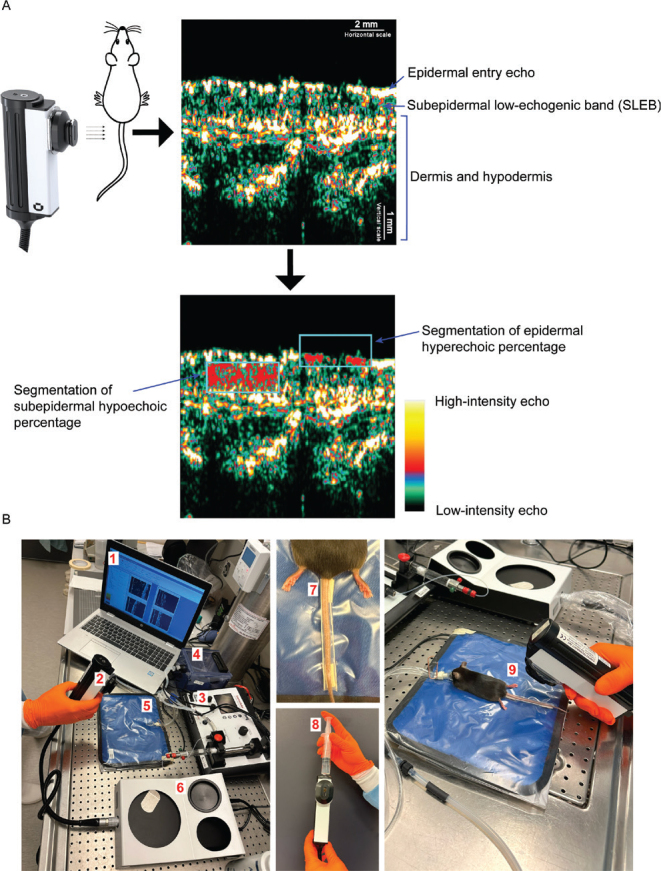
**Schematic of ultrasound analysis and imaging setup.** (A) The epidermal entry echo was defined as the first hyperechoic region of the skin. The subepidermal low-echogenic band (SLEB) was defined as the hypoechoic area located beneath the epidermal entry echo, extending down to the more hyperechoic dermis. The schematic illustrates how the rectangular region of interest (ROI) was positioned for the segmentation of subepidermal hypoechoic percentage and epidermal hyperechoic percentage. For segmentation, intensity values of 0–30 were classified as hypoechoic and values of 150–255 as hyperechoic. (B) Setup and procedure for HFUS image acquisition. (1) Computer running the image acquisition and analysis software. (2) A 20 MHz ultrasound probe, enabling B-scans at 60 × 150 μm resolution and 13 mm penetration. (3) Isoflurane anaesthesia system. (4) Chamber for anesthetizing mice. (5) Heating pad to maintain stable body temperature. (6) DermaScan HFUS unit. (7) Disposable fixation rail, devised by cutting a 1 mL pipette tip in half, used to stabilize the mouse tail. (8) Fluid was added to the probe chamber before imaging, followed by the application of ultrasound gel to the probe slit. (9) Final image acquisition with the probe gently placed perpendicular to the dorsal tail surface.

**Fig. 2:**
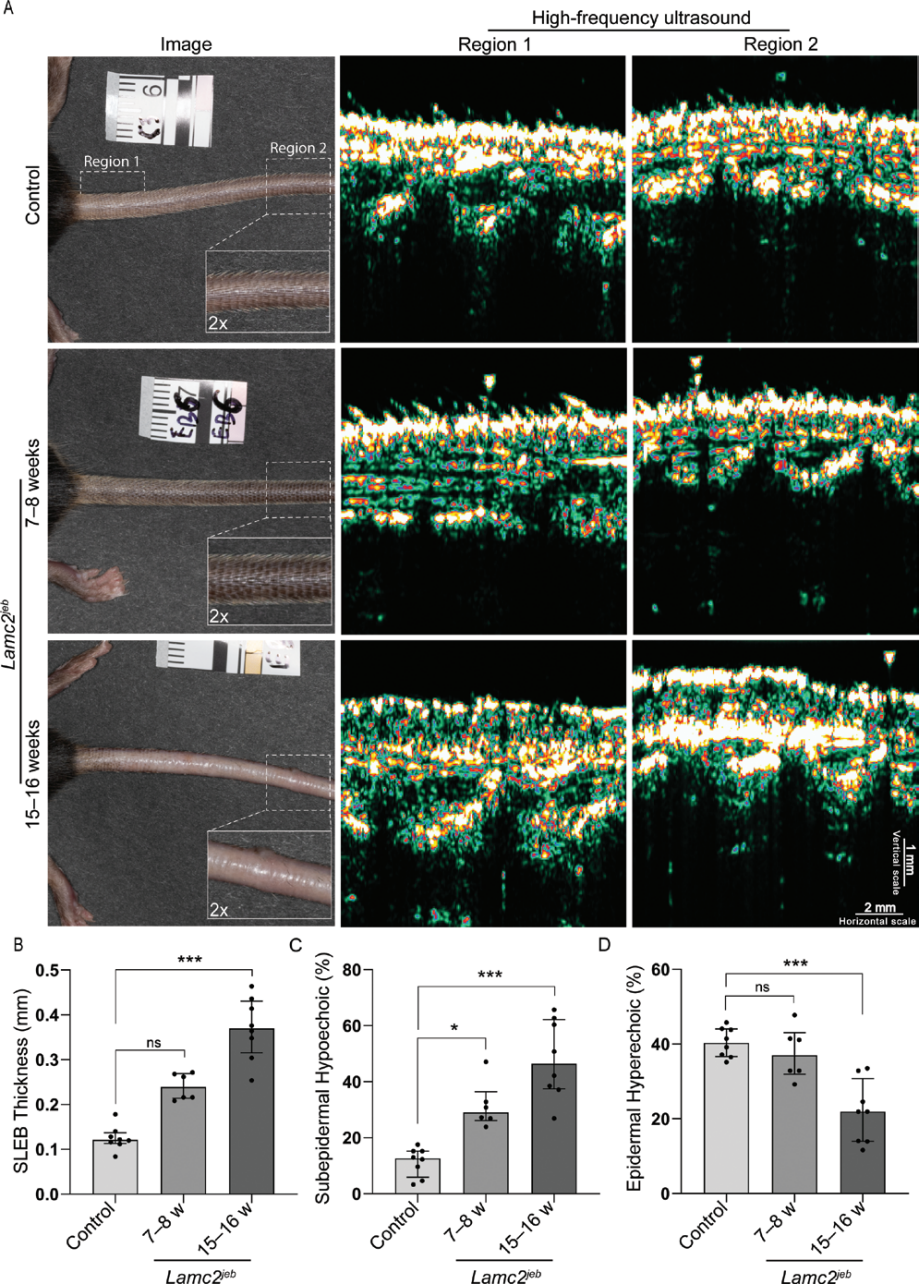
**Quantification of high-frequency ultrasound (HFUS) images.** (A) Representative cases from each group, including photographs showing tail morphology and locations of Regions 1 and 2 used for HFUS imaging. HFUS images revealed a more pronounced subepidermal low-echogenic band (SLEB) and a less homogeneous epidermis in the 15–16-week *LAMC2^jeb^* group than in the age-matched wild-type controls and the 7–8-week *LAMC2^jeb^* group. (B–D) Scatter plots with bars show the median with interquartile range for SLEB thickness, subepidermal hypoechoic percentage, and epidermal hyperechoic percentage. For all analyses, each measured region was considered an independent sample (controls age-matched to the 15–16-week group, *n* = 8; 7–8-week *LAMC2^jeb^* group, *n* = 6; and 15–16-week *LAMC2^jeb^* group, *n* = 8). Statistical testing was performed using the Kruskal–Wallis test, with multiple comparisons between the controls and *LAMC2^jeb^* groups. Dunn’s test was used to correct for multiple comparisons. Significance levels: ns > 0.05, *p ≤ 0.05, **p ≤ 0.01, ***p ≤ 0.001. Statistical analyses were performed using GraphPad Prism version 10.4.1 (https://www.graphpad.com/).

A DermaScan HFUS unit (Cortex, Denmark) with a 20 MHz probe was used. A schematic is provided in **Fig. 1**A. All ultrasound images were acquired using a standardized gain profile (DermaScan preset gain profile 3, gain level 13). HFUS image quantification was performed using DermaScan analysis software (version 1.10.0.4, Cortex, Odense, Denmark). SLEB thickness was measured in A-scan mode by averaging 5 separate vertical measurements (peak-to-peak) from the epidermal entry echo to the first dermal peak (2). Two tail regions were defined: Region 1 at the tail base and Region 2, located approximately 3 cm from the base (**Fig. 2**A). Fig. 1B provides a schematic of the materials used and practical procedures.

B-scan mode using a rectangular region of interest (ROI; Preset shape 4 = area 1.51 mm²) was used for segmentation. Segmentation was repeated twice at different locations in the same image, with mean percentage reported. For subepidermal hypoechoic percentage, the ROI’s upper boundary was aligned with the epidermis, using threshold values of 0 and 30 (8). For epidermal hyperechoic percentage, the ROI was aligned with the lower boundary of the epidermal entry echo with threshold values of 150 and 255 (see Fig. 1A).

For animals imaged using HFUS, the proximal and distal tail regions corresponding to HFUS Regions 1 and 2 were excised and fixed in neutral-buffered formalin. H&E-stained slides were imaged using an AxioScope A.1 microscope (Zeiss, Oberkochen, Germany). Five non-overlapping representative areas were selected from each sample region at 50× magnification. Two independent assessors, blinded to image identity, scored epidermal detachment, immune cell infiltration, and epidermal architecture using a 0–3 scale: grade 0, no changes; grade 1, mild; grade 2, moderate; and grade 3, extensive (adapted from Wang et al. [9]). Scores were averaged per assessor and region.

Fig. 1 illustrates the ultrasound imaging and analysis setup. Two loops of 100 images were recorded per mouse in Regions 1 and 2 (Fig. 2A). The highest-quality frame from each loop, ensuring the epidermal entry echo remained as horizontal as possible, was selected for subsequent analysis.

Three *LAMC2^jeb^* mice aged 7–8 weeks with subtle JEB-related features (primarily on ears and not tail), 4 *LAMC2^jeb^* mice aged 15–16 weeks with a distinctive JEB phenotype (e.g., hair loss, mild ulceration, and blistering on the tail), and 4 wild-type C57BL/6 mice (15–16 weeks old, age-matched to the second JEB group) were imaged. Visual inspection of HFUS images (Fig. 2A) showed that wild-type mice typically exhibited a homogeneous epidermal entry echo and negligible SLEB, suggesting tight dermal-epidermal attachment. In contrast, 7–8-week *LAMC2^jeb^* mice displayed a similarly homogeneous epidermal entry echo but also consistently showed a discernible SLEB. These differences were more pronounced in the 15–16-week *LAMC2^jeb^* group, which generally exhibited a more irregular epidermal entry echo and a better defined and thicker SLEB with hypoechoic areas completely separating the dermis and epidermis.

Quantification of SLEB thickness (Fig. 2B) revealed a general increase in *LAMC2^jeb^* mice compared with wild-type controls, with the 15–16-week group exhibiting the highest values. The 7–8-week group had a non-significant increase in median SLEB thickness compared with the controls (0.239 vs 0.121 mm, *p* = 0.06), whereas the 15–16-week group exhibited a highly significant difference (0.370 vs 0.121 mm, *p* < 0.001). Quantification of the subepidermal hypoechoic percentage (Fig. 2C) yielded similar results: compared with controls, the 7–8-week group displayed a significant increase in median percentage (29.0 vs 16.6%, *p* = 0.04), and the 15–16-week group showed a highly significant increase (46.5 vs 16.6%, *p* < 0.001). Quantification of the epidermal hyperechoic percentage (Fig. 2D) showed no statistically significant difference between the controls and 7–8-week *LAMC2^jeb^* group (37.0 vs 40.3%, *p* = 0.78). However, the 15–16-week *LAMC2^jeb^* mice exhibited a highly significant reduction (30.8 vs 40.3%, *p* < 0.001) compared with the controls.

Two control mice and 2 *LAMC2^jeb^* mice (1 mouse with extensive hair loss and tail ulcerations and 1 mouse with no tail ulceration and less hair loss) (Fig. S1), all age-matched and 15–16 weeks old, were used for H&E histological assessment. HFUS and H&E images from matched regions exhibited visual structural correlations (Fig. S2). The average histology scores from each assessor, along with corresponding HFUS quantification, are presented in **Table I**. Higher epidermal detachment and immune cell infiltration generally coincided with increased SLEB thickness and hypoechoic areas, whereas greater epidermal architecture scores coincided with reduced hyperechoic percentages. Regional disparities were observed in the less severe *LAMC2^jeb^* specimens. Histopathology showed elevated epidermal detachment and immune infiltration in Region 2, whereas HFUS detected higher SLEB thickness and subepidermal fluid in Region 1.

**Table I:** Comparison between high-frequency ultrasound (HFUS) quantification and histopathological scoring performed by 2 blinded investigators. Each histopathological score was averaged across 5 images per assessor and region

Item			Histopathology scoring (0–3)			HFUS Measurements		
		Scorer	Epidermal detachment	Immune cell infiltration	Epidermal architecture	SLEB thickness (mm)	Subepidermal hypoechoic (%)	Epidermal hyperechoic (%)
Control 1	Region 1	1	0	0.2	0.2	0.122	3.32	45.8
		2	0	0.4	0.2			
	Region 2	1	0	0	0.2	0.084	12.3	36.5
		2	0	0.1	0.1			
Control 2	Region 1	1	0	0	0.6	0.12	17.6	44.1
		2	0.6	0.2	0.6			
	Region 2	1	0	0	0	0.128	4.65	39.2
		2	0	0	0			
LAMC2^jeb^ (severe)	Region 1	1	1.4	2.4	2	0.414	62.7	21.9
		2	1.4	2.2	1.6			
	Region 2	1	3	2.8	2.6	0.464	65.7	14.1
		2	3	3	2.6			
LAMC2^jeb^ (less severe)	Region 1	1	0.8	0.6	1	0.304	38.5	33.5
		2	0.6	0.4	0.2			
	Region 2	1	1.6	1.2	0.8	0.254	26.9	32.9
		2	1.8	1.2	0.8			

## DISCUSSION

The findings presented here indicate that HFUS assessment, including measurement of SLEB thickness, subepidermal hypoechoic percentage, and epidermal hyperechoic percentage, can monitor disease progression from early subclinical changes to significant structural disruptions in a murine model of JEB.

The corresponding pathological changes underlying the observed alterations in HFUS metrics are not fully understood but may be related to inflammation, a well-described feature of EB wounds and the surrounding skin (1, 10). Inflammation often results in tissue oedema, which may correspond to the increase in hypoechoic signals detected by HFUS, supported by previous reports showing a correlation between SLEB thickness and inflammatory activity in skin diseases (3–6). The basis for the more irregular epidermal entry echo and decreased epidermal echogenicity is unclear. Potential explanations include that an altered epidermal architecture due to hyperproliferation causes less echogenicity or that the differences observed are related to the hair loss observed in severe cases, as differentiation between the epidermis and such thin hair strands is challenging using 20 MHz resolution (11). Given these limitations, including the inability of histopathology to measure fluid content, targeted studies are needed to define the structural basis of HFUS alterations in EB. Future experimental studies in the *LAMC2^jeb^* model should also examine anatomical site-specific differences, for example by inducing controlled test lesions in shaved back skin and comparing their HFUS profiles with those of naturally blistering tail skin.

HFUS protocols could enable longitudinal monitoring of EB in preclinical and clinical settings, allowing simultaneous real-time assessment of multiple areas to evaluate disease severity and treatment results. Future research should focus on studying HFUS metrics and their correlation to clinical outcomes in diverse populations of EB patients.

## Supplementary Material


