# Supplementary material for: High-frequency Ultrasound Reveals Epidermal-dermal Structural Alterations in an Experimental Model of Epidermolysis Bullosa
Source: Acta Derm Venereol. 2026 Feb 17;106:44438. doi: 10.2340/actadv.v106.44438 (PMC12924092; doi:10.2340/actadv.v106.44438)
Supplement: Supplementary file 1 [file ActaDV-106-44438-s1.pdf]

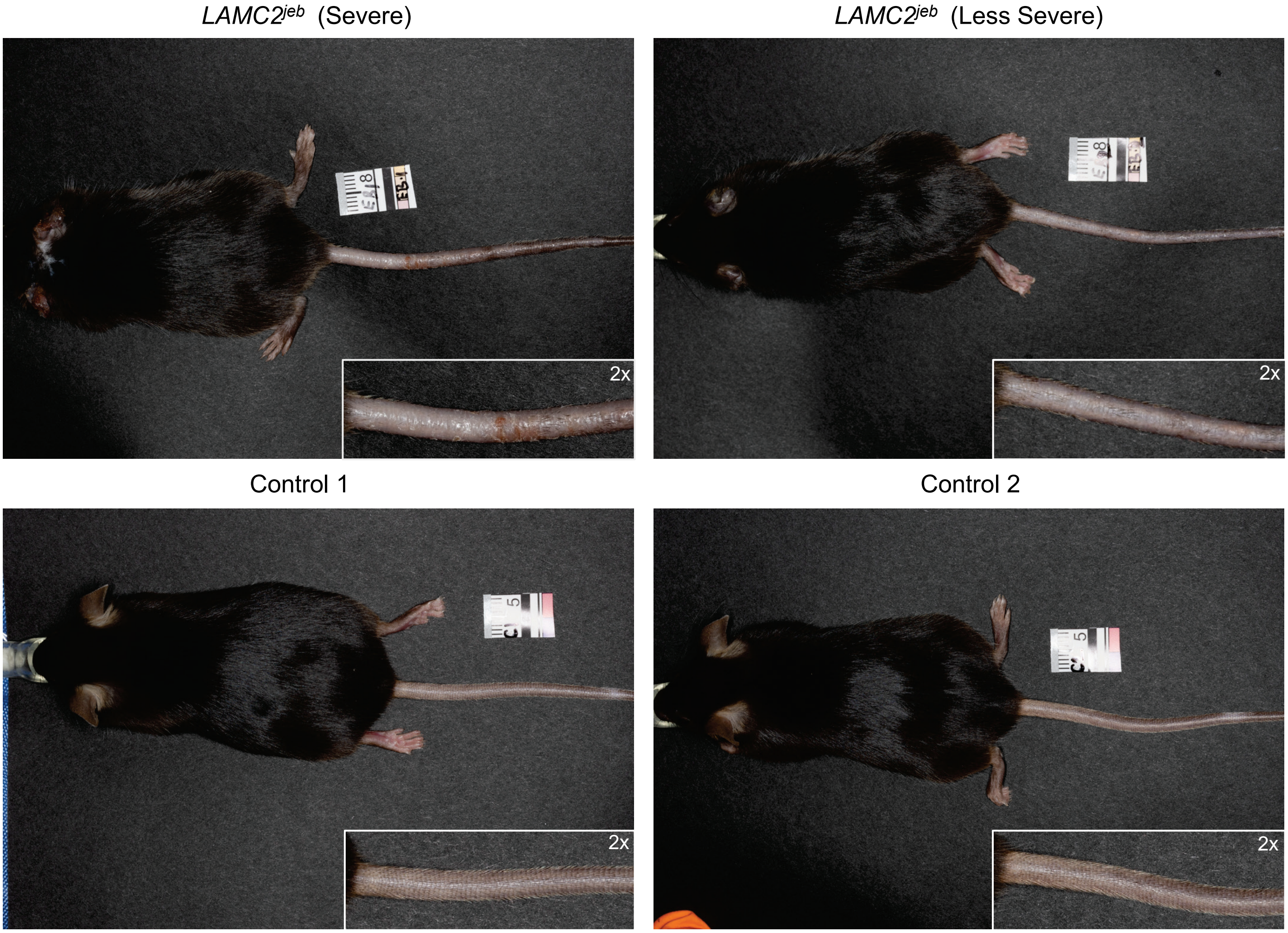

Supplementary Figure 1. Photographs of mouse specimens utilized for comparative histopathological and high-frequency ultrasound analyses. Two *LAMC2*^{jeb} mice aged 15–16 weeks were examined: one exhibiting a severe junctional epidermolysis bullosa (JEB) phenotype characterized by extensive hair loss and tail ulcerations, and another presenting a milder phenotype with less hair loss and no tail ulcerations. Age-matched wild-type control mice showed no visible pathological features. Inserts represents a 2x digital zoom of the proximal tail. Images were captured with a Canon EOS R7 camera (Canon, Tokyo, Japan) equipped with a Canon EF-S 60/2.8 Macro USM lens (Canon, Tokyo, Japan). Photographic parameters, including distance and camera settings, were standardized across all images.

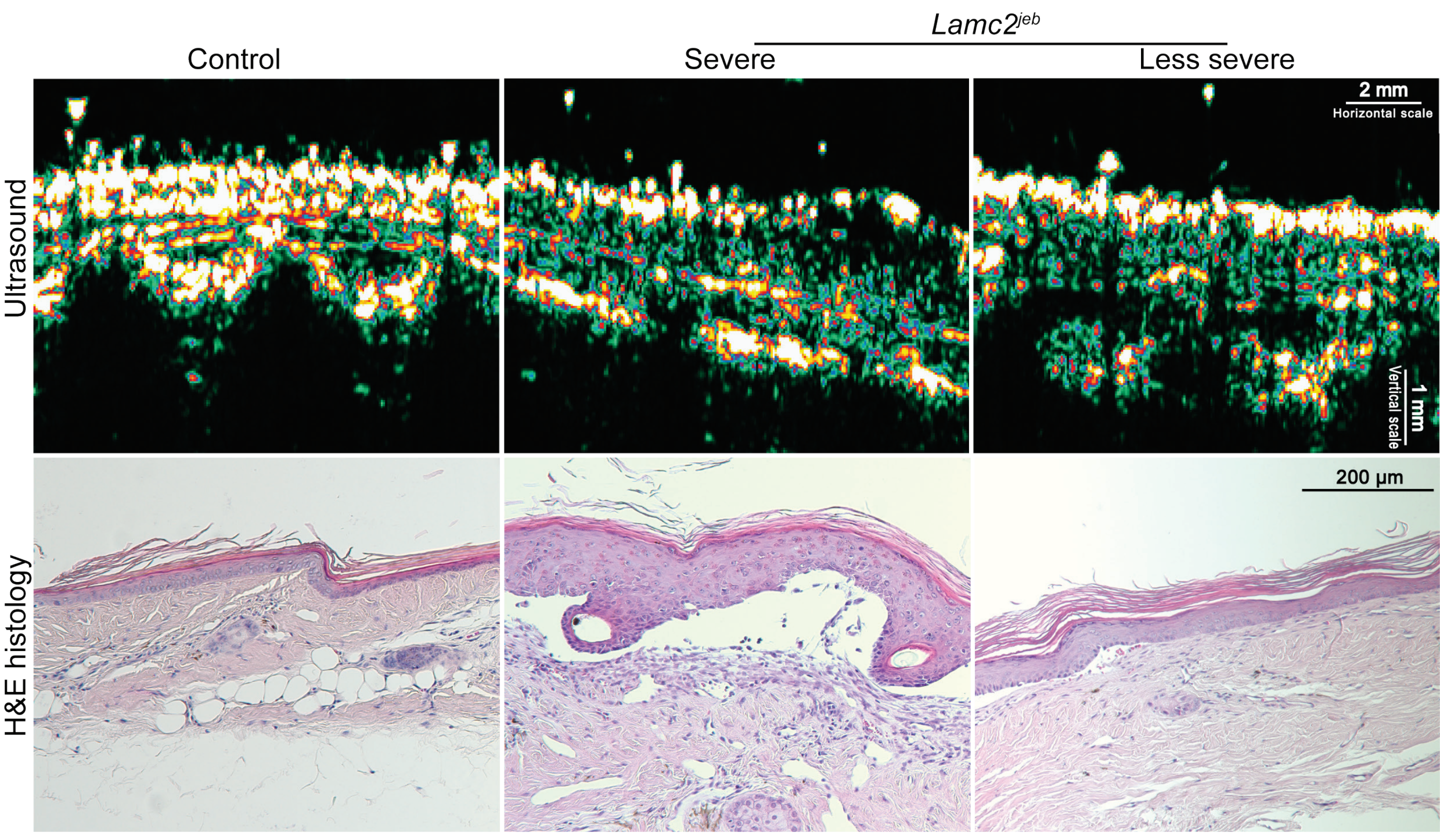

Supplementary Figure 2. Correspondence of high-frequency ultrasound (HFUS) and histopathology. Representative HFUS images and corresponding hematoxylin and eosin (H&E)-stained sections from matched regions in 15–16-week-old, age-matched mice: two *LAMC2^{jeb}* mice (one with a severe phenotype and one with a less severe phenotype) and one wild-type control mouse. The wild-type controls generally displayed a homogeneous epidermal entry echo on HFUS and a corresponding intact epidermal-dermal junction with no signs of immune cell infiltration on H&E. In the severe junctional epidermolysis bullosa (JEB) phenotype, HFUS generally showed an irregular epidermal entry echo and a prominent subepidermal low-echogenic band (SLEB). This corresponded to marked epidermal hyperplasia, near-complete detachment at the epidermal-dermal junction, and prominent subepidermal immune cell infiltration on H&E. In the less severe JEB phenotype, HFUS generally showed a thicker but uniform epidermal entry echo and a less pronounced SLEB, corresponding to mild epidermal hyperplasia with only focal dermal detachment and visible but less pronounced immune infiltration on histology.
